# Validation of the Hospital Survey on Patient Safety Culture 2.0 in Italian Hospitals: A Cross‐Sectional Study of Healthcare Personnel Perceptions

**DOI:** 10.1111/jan.16770

**Published:** 2025-01-26

**Authors:** Annamaria Bagnasco, Gianluca Catania, Michele Tancredi Loiudice, Tommaso Bellandi, Bruno Cavaliere, Sara Carzaniga, Flavia Cardinali, Milko Zanini, Loredana Sasso, the Working Group Study, Nicola Pagnucci, Michela Calzolari, Francesca Napolitano, Davide Ulivieri, Spartaco Mencaroni, Luca Lavazza

**Affiliations:** ^1^ Dipartimento Scienze Della Salute Università degli Studi di Genova Genova Italy; ^2^ UOS Rischio clinico e Sicurezza delle cure Agenas ‐ Agenzia Nazionale per i Servizi Sanitari Regionali Roma Italy; ^3^ Direttore UOC Sicurezza del Paziente Azienda USL Toscana Nord Ovest Lucca Italy; ^4^ Direttore Professioni Sanitarie, Ospedale IRCCS Policlinico San Martino Genova Italy

**Keywords:** patient safety, safety culture, safety management

## Abstract

**Aim(s):**

To adapt and validate the HSOPS 2 instrument for the Italian context and to describe the current patient safety culture amongst healthcare personnel working in Italian hospitals.

**Design:**

Cross‐sectional study.

**Methods:**

We adapted and validated the HSOPS 2 instrument following the COSMIN guidelines: we performed a *forward‐backward translation*, calculated the content validity index, evaluated face validity, acceptability (percentage of participants responding to all items on the questionnaire and to every specific item), construct validity (confirmatory factor analysis), and internal consistency (Cronbach's alpha for each dimension). We then performed a cross‐sectional study following the guidelines of the original instrument: we categorised the responses into “positive,” “negative,” and “midpoints.” For each dimension we calculated the average percentage of positive responses. We repeated this process, dividing the responses by various sample characteristics (e.g., profession), and compared them using the chi‐square test. Data were collected between April and November 2023.

**Results:**

A total of 633 hospital personnel participated in the survey, and 473 completed the questionnaire in its entirety. The dimensions of “teamwork”, “supervisor”, “manager”, or “clinical leader support”, and “communication about error” emerged as dimensions with higher percentages of positive responses, while those that received lower percentages were “hospital management support for patient safety”, “staffing and work pace”, and “response to error”. We identified statistically significant differences in many dimensions between gender, profession, and clinical inpatient units.

**Conclusions:**

These findings provide a comprehensive overview of challenges and opportunities within the healthcare sector as regards patient safety culture and can inform the development of targeted interventions aimed at improving patient safety across healthcare organisations.

**Implications for the Profession and/or Patient Care:**

Proper assessment of safety culture, one of the main indicators of patient safety, can inform the development of effective strategies and interventions to enhance patient safety.

**Impact:**

*What problem did the study address?*

- To effectively assess patient safety culture, it is essential to use valid and reliable tools.
- It is crucial to proactively assess patient safety culture in hospital personnel, whether employed in clinical units, in management, or in support services, to develop initiatives aimed at improving patient safety.

*What were the main findings?*

- The use of the adapted and validated version of the HSOPS 2 will produce valid and reliable evidence on patient safety culture.
- Perception of patient safety culture differs amongst respondents according to gender, profession, clinical setting.
- The dimensions of “hospital management support for patient safety”, “staffing and work pace”, and “response to error” were identified as those with the greatest need for improvement.

*Where and on whom will the research have an impact?*

- Patient safety heavily impacts care at every level; therefore, this study could have an impact on healthcare organisations as well as healthcare workers, patients, and their families.
- By making available an instrument that can contribute to a proper assessment of patient safety culture, this study might contribute to the development of appropriate strategies and targeted interventions to improve patient safety, quality of care and satisfaction while decreasing adverse events and related costs.

**Reporting Method:**

The COSMIN guidelines were used for the validation of the instrument; the STROBE reporting guidelines were used for the cross‐sectional study.

**Patient or Public Contribution:**

No patient or public contribution.

**Summary**

- What does this paper contribute to the wider global clinical community?
  ○ Promotes global harmonisation of patient safety culture assessment: By contributing to the growing body of validated translations of the HSOPS 2 instrument, this study supports the standardisation of patient safety culture evaluations worldwide, enabling meaningful international comparisons and fostering collaborative efforts to improve healthcare safety globally.
  ○ Framework for tailored interventions and policy development: By identifying variations in safety culture perceptions based on gender, profession, and clinical units, the paper provides a data‐driven foundation for designing customised interventions, shaping policies, and optimising patient safety practices worldwide.
- What is already known?
  ○ Patient safety culture is a key factor influencing healthcare quality, staff performance, and patient outcomes.
  ○ The Hospital Survey on Patient Safety Culture (HSOPS) is a widely recognised tool for assessing safety culture in hospitals.
  ○ Previous studies identified gaps in patient safety culture, such as staffing issues, communication barriers, and organisational support, which impact safety practices.
- What this paper adds?
  ○ Provides a validated Italian version of the HSOPS 2 instrument, contributing to the global harmonisation of patient safety culture assessments.
  ○ Highlights variations in safety culture perceptions based on gender, profession, and clinical units, revealing disparities in attitudes and practices.
  ○ Identifies specific areas for improvement, such as staffing, hospital management support, and response to errors, offering insights for targeted interventions.
- Implications for practice/policy
  ○ Supports the adoption of standardised tools to measure and monitor patient safety culture across diverse healthcare systems globally.
  ○ Provides data‐driven evidence to inform the development of tailored interventions addressing identified gaps in staffing, communication, and management support.
  ○ Encourages policies that promote continuous learning, transparent error reporting, and staff engagement to foster a sustainable culture of safety in healthcare settings.

## 1 Introduction

In 2016, a series of annual meetings of health ministers from around the world was initiated to bring together international experts and policymakers for effective collaboration in patient safety (WHO 2016). The UN World Health Assembly endorsed the establishment of a World Patient Safety Day on 17 September each year to further strengthen awareness and stimulate combined action for safer care (WHA 2019).

Over the past decades, the field of patient safety has expanded and evolved in several directions. Early studies regarding this phenomenon focused on understanding the dimensions and different types of adverse events and the varying degrees of harm they could cause to patients (Carayon, Xie, and Kianfar 2014; Wears, Kathleen, and Van Rite 2016). International studies analysed in the systematic review by De Vries et al. (2008) showed that adverse events during hospitalisation affect almost 1 in 10 patients and that these are preventable in almost half of the cases. More recent evidence confirms that more than 15% of hospital spending and activities in OECD countries can be attributed to the treatment of patients who suffer harm from adverse events due to poor safety, many of which are therefore preventable (Gautam 2017; Slawomirski, Auraaen, and Klazinga 2017).

In Italy, according to a retrospective study involving five large hospitals, the incidence of adverse events was 5.2%, with a prevalent distribution in the medical area (37.5%) and an overall preventability of 60% (Tartaglia et al. 2012). Similarly, the study by Sommella et al. (2014) retrospectively identified an incidence of adverse events of 3.3%, which mostly took place in the medical area (71.7%), in the surgical area (19.6%), and in the intensive care unit (8.7%). More recently, a large multicentre retrospective study in the emergency departments nationwide performed an analysis of hospitalisations and visits to the emergency department caused by adverse events related to prescribed drugs (Lombardi et al. 2020). Of the 61,855 adverse drug events reported, 30.6% resulted in hospitalisation, of which 47% were preventable.

Traditionally, clinicians present complications at surgical morbidity and mortality (M&M) conferences, and the Agency for Healthcare Research and Quality (AHRQ) Patient Safety Indicators (PSIs) use inpatient administrative data to identify certain adverse outcomes. Although both methods are used to identify adverse events and inform quality improvement efforts, these two methods do not overlap: self‐reported adverse events by surgeons in M&M and the PSIs are complementary approaches to identifying complications. Both case‐finding processes should be used to inform quality improvement efforts (Anderson et al. 2018).

In addition to the identification and description of adverse events to understand their nature and causes, the attention of researchers in the field of patient safety has also focused on the narrower concept of patient safety and its promotion, increasingly considering it to have a major impact on patient safety (Pacenko et al. 2024; Lorenzini et al. 2017). The concept of patient safety culture refers to the shared values, beliefs, and norms about the importance of patient safety within a healthcare organisation, the interaction within the staff and between the staff and the patients, and the attitudes and behaviours related to patient safety (AHRQ 2019a, 2019b). Measuring patient safety culture is critical for identifying systemic issues and guiding targeted interventions, while comprehensive assessments offer insights into strengths and weaknesses, fostering continuous improvement and reducing risks in healthcare organisations (AHRQ 2022a, 2022b).

## 2 Background

The recent shift of focus of interest regarding patient safety highlighted the importance of understanding the role played by human and organisational factors, such as leadership, teamwork, and communication, in contributing to and preventing adverse incidents (Waterson and Catchpole 2016).

In particular, the organisational factor of ‘safety culture’, has attracted much interest and discussion and began to be considered amongst the main indicators of patient safety (Singer and Vogus 2013; Stelfox et al. 2006), to the point of becoming one of the seven pillars of the Global Patient Safety Action Plan 2021–2030 developed by the World Health Organisation (WHO 2021).

The use of the term ‘safety culture’ first emerged following the Chernobyl nuclear accident. Since then, it has been used as a way of understanding accidents in a wide variety of sectors, including aviation, oil and gas and, more recently, the health sector (Vincent and Amalberti 2016). The aviation industry has been the most responsive sector in this field and has thrived due to positive underlying attitudes; similarly, the oil and gas industry has improved due to its more systematic and calculative approach. Both converged on implementing strategies that aim to recognise and learn from mistakes to become more proactive towards safety (Vincent and Amalberti 2016).

In light of this realisation, although the health system is still at the early stages of growth on safety culture issues, improving patient safety remains one of the most pressing health issues for public awareness and further political action. In the healthcare settings, safety culture appears to be associated with issues such as missed care, adverse patient safety events, quality of care, and burnout (Camacho‐Rodríguez et al. 2022).

The AHRQ recognised the complex nature of patient safety culture as something that permeates multiple levels in organisations, including the unit, department, organisation, and system levels. To develop a more comprehensive approach to the assessment of patient safety across healthcare systems and staff, it is therefore essential to assess patient safety culture. According to the AHRQ, patient safety culture can be measured by determining what is rewarded, supported, expected, and accepted in an organisation as it relates to patient safety (AHRQ 2023).

Recognising the limitations of relying solely on administrative data and on staff‐reported incidents, an increasing number of OECD countries are incorporating alternative data sources, such as staff‐reported safety culture. To do so, various questionnaires and instruments to assess and monitor staff‐reported patient safety culture have been developed (de Bienassis and Klazinga 2022). These initiatives underscore the growing recognition of the multifaceted nature of patient safety and the need for comprehensive approaches to address it. The insights uncovered from data collected directly from various staff members play a pivotal role in preventing and evaluating patient safety incidents as well as in addressing them in appropriate and effective manners (Azyabi, Karwowski, and Davahli 2021).

A recent systematic review (Azyabi, Karwowski, and Davahli 2021) identified several instruments used to assess patient safety culture, in particular: the Hospital Survey on Patient Safety Culture (HSOPS), the Safety Attitudes Questionnaire (SAQ), the Patient Safety Climate in Health Care Organisations (PSCHO), the Modified Stanford Instrument (MSI‐2006), and the Scottish Hospital Safety Questionnaire (SHSQ). All these instruments focus on factors such as teamwork, organisational and behavioural learning, reporting of errors and safety awareness, gender and demographics, work experience, and staffing levels that were perceived as significantly impacting patient safety (Azyabi, Karwowski, and Davahli 2021). The HSOPS, developed by the AHRQ (AHRQ 2004), is one of the most utilised and comprehensive tools to assess patient safety culture amongst healthcare professionals working in hospitals. In 2010, this questionnaire was validated and used in the Italian context (Bagnasco et al. 2011) to assess patient safety culture amongst healthcare professionals working in Italian hospitals. Since then, the AHRQ has updated the instrument over the years, producing the latest version, the HSOPS 2, in 2019, that has yet to be implemented in the Italian context (AHRQ 2019a, 2019b).

Integrating the findings from such validated instruments into broader healthcare system evaluation and improvement efforts holds immense potential to bolster patient safety outcomes and enhance organisational effectiveness. Conducting studies on patient safety culture with validated tools like HSOPS 2 is crucial to ensuring the reliability and validity of the findings, thereby facilitating informed decision‐making and targeted interventions for safer healthcare services to foster international comparisons and cooperation.

This study is part of a wider collaborative project between the public institutions of the authors, created to validate and disseminate the Italian versions of HSOPS 2 and OECD Patient‐Reported Incident Measures (PRIMs), as well as to explore correlations between patient safety logistics, safety culture, patient‐reported incident measures (PREMS), and patient outcomes (PROMS).

## 3 The Study

### 3.1 Aim(s)

The aim of the study was to adapt and validate the HSOPS 2 instrument to the Italian context and to describe the current patient safety culture in a sample of hospital personnel working in Italian hospitals.

### 3.2 Secondary Objective

Secondary objective of the study was to identify differences between participants' characteristics and patient safety culture.

## 4 Methods

### 4.1 Design, Study Setting, and Sampling

This was a cross‐sectional study that utilised an online survey incorporating the HSOPS 2 instrument. We selected two hospitals in two Italian regions that belonged to the participating institutions and are part of the Italian National Health Service (NHS); therefore, they were involved as a test bed for this project. Given that in Italy there is national legislation with specific objectives and requirements for patient safety (Bellandi et al. 2017), we assume that the working conditions are comparable in the two settings, even though some activities for patient safety are further articulated at the regional and local level. The study population consisted of hospital personnel of these two participating hospitals who have expressed a willingness to participate in the study and that signed the informed consent.

#### 4.1.1 Sample Size

Following the recommendations of Tabachnick and Fidell (2012), who recommend having at least 10 participants for each item with a minimum of 200 to 300 subjects and estimating a dropout rate of 20%, we assumed a minimum sample size of 460 participants amongst the staff of the participating centres.

#### 4.1.2 Inclusion and/or Exclusion Criteria

*Inclusion criteria*

- All hospital personnel currently in service at the participating hospitals during the study period.
- Informed consent to participation.

*Exclusion criteria*

- Hospital personnel currently in service at day hospital (a planned admission, or cycle of admissions limited to a single part of the day and does not, therefore, cover the entire 24‐h period from the time of admission; it provides multi‐professional and/or multi‐specialist services, which require a performance time that is markedly different from that required for a normal outpatient service), day surgery (enables the admission and management of patients with low‐complexity surgical conditions in which surgery and discharge take place on a single day), outpatient context of participating hospitals.
- Hospital personnel currently in service at community and territorial services of participating healthcare organisations (including but not limited to social and health districts, health homes, assisted living facilities, residential facilities for the disabled, day care centres, mental health service residences, rehabilitation centres).
- Hospital personnel who have already completed the questionnaire.

### 4.2 Definition of Patient Safety Culture

The term patient safety culture is widely used in healthcare research to describe the shared values, beliefs, and norms about the importance of patient safety within a healthcare organisation, how staff interact with each other and with patients, and the attitudes and behaviours related to patient safety (AHRQ 2019a, 2019b).

### 4.3 The Instrument

The HSOPS 2.0 (AHRQ 2019a, 2019b) questionnaire aims at assessing a hospital's patient safety culture. The questionnaire is an update of the original SOPS Hospital Survey (1.0) published by AHRQ in 2004 (AHRQ 2004). The HSOPS 2.0 consists of 40 questions divided into eight single measures (one question that investigates the number of patient safety events reported by the participant; one question related to the overall assessment of patient safety in one's own unit/work area; six questions related to the participant's characteristics such as job position, unit/work area, assignment in the hospital, assignment in the unit/work area, working hours, interaction with patients). 32 questions grouped into 10 composite measures assessing the areas of patient safety culture (teamwork; staffing and work pace; organisational learning‐continuous improvement; response to error; supervisor, manager, or clinical leader support for patient safety; communication about error; communication openness; reporting patient safety events; hospital management support for patient safety; handoffs and information exchange).

These 32 questions use a five‐point Likert scale (from 1: “Strongly disagree” to 5: “Strongly agree”) or frequency scales (from 1: “Never” to 5: “Always”) and include the response option 6: “Not applicable or Don't know”. The questionnaire includes a final section for open comments.

The instrument showed good reliability, with Cronbach's alpha ≥ 0.7 for six dimensions, ≥ 0.8 for three dimensions and a single dimension that obtained a Cronbach's alpha at the limit of acceptability with a value of 0.67.

### 4.4 Data Collection and Data Analysis

We collected and analysed data obtained by the online administration (Lymesurvey platform) of the HSOPS 2 at a single time point. Data were collected between May and November 2023.

The staff from the participating centers were invited to participate in the study through multiple recruitment methods, including email invitations and informational posters displayed within the centers. These posters contained a link to an online questionnaire and provided detailed information about the study, including its objectives, procedures, and ethical considerations. The study's information sheet and the informed consent form were also made available through these materials. Participants were only included in the study after they gave their consent by electronically signing the consent form.

#### 4.4.1 Validation of the Instrument

We used the *forward‐backward translation* method to translate HSOPS 2 from the English language. We asked two different Italian mother‐tongue translators with a good knowledge of the English language to independently translate the questionnaire. The two translations were reconciled, and the final version was translated back into English by an English mother‐tongue translator with a good knowledge of the Italian language. We submitted the translated version of the questionnaire using an online platform (Lymesurvey) to a group of experts in April 2023 to validate its content. Following the recommendation from Polit, Beck, and Owen (2007), we calculated the Content Validity Index for each item (I‐CVI) and for the scale (S‐CVI), and we considered acceptable I‐CVI ≥ 0.78 and S‐CVI ≥ 0.90. We then submitted the resulting version of the questionnaire to a group of possible participants and asked them to evaluate if the items were difficult to understand, confusing, contained difficult words, contained offensive words, or needed to be rephrased. Answers were qualitatively analysed.

Finally, we evaluated acceptability, construct validity, and internal consistency (Tabachnick and Fidell 2012). As regards acceptability, it was assessed in terms of compliance for the scale (percentage of healthcare workers responding to all items on the questionnaire) and for each item on the questionnaire (percentage of healthcare workers responding to the specific item). In addition, we evaluated the floor and ceiling effects for each item. To evaluate the construct validity, we performed a confirmatory factor analysis (CFA), based on the construct defined in the original development of the HSOPS 2 (AHRQ 2019a, 2019b; Sorra et al. 2019). Firstly, we evaluated the distribution of the data (Shapiro–Wilk test), the adequacy of the correlation matrix (Barlett's test), considering *p* values ≤ 0.05, and the sampling adequacy (Kaiser‐Meyer‐Olkin test), considering acceptable values > 0.5. Performing the CFA, we considered acceptable comparative fit index (CFI) values between 0.90 and 1, goodness of fit index (GFI) values > 0.90, normed chi‐square values (*χ* ^2^/df) < 5, root mean square error of approximation (RMSEA), and standardised root mean square residual (SRMR) values < 0.80 (Kline 2023; Shevlin and Miles 1998). Regarding the internal consistency, we evaluated Cronbach's alpha for each dimension derived from the factor analysis. We considered acceptable values ≥ 0.7, good values ≥ 0.8, and excellent values ≥ 0.9 (Streiner, Norman, and Cairney 2024).

#### 4.4.2 Cross‐Sectional

Following the guidelines of the original instrument, we categorised the responses for each item into “positive,” “negative,” and “midpoints”. We did this differently for positively worded items (e.g., “In this unit, we work together as an effective team”) and negatively worded items (e.g., “We have patient safety problems in this unit”). For positively worded items, positive responses were considered those from participants who answered “Strongly agree”/“Agree” or “Always”/“Most of the time”. For negatively worded items, positive responses were considered those from participants who answered “Strongly disagree”/“Disagree” or “Never”/“Rarely”, because a negative answer on a negatively worded item indicates a positive response. Both for positively and negatively worded items, the “midpoints”, or neutral responses, consisted in the answers “Neither agree nor disagree” (AHRQ 2019a, 2019b). Then, for each dimension confirmed by the factor analysis, we measured the percentage of positive responses over the total respondents for each item in that dimension. We then calculated the average percentage of positive responses for each dimension.

After we did this calculation on the total sample, we repeated the same process but divided the responses by the various sample characteristics (gender, profession, clinical inpatient unit) that were coded in order to finally compare them using the chi‐square test to identify any statistically significant (*p* ≤ 0.05) differences.

We performed all the analysis using Jamovi ver. 2.4.8, except for the CFA, for which we used Jasp ver. 0.18.1.

### 4.5 Ethical Considerations

The study was conducted in accordance with the Declaration of Helsinki (Fortaleza version 2013) and in accordance with current regulations on clinical trials and good clinical practice. The promoter of the study undertakes to protect sensitive personal data of the participants involved in the study in accordance with the provisions of European legislation (EU Regulation GDPR 2016/679). The absolute voluntary nature of participation in the study was guaranteed, as well as the possibility of not giving one's consent and of withdrawing from the study at any time of its execution. All data were anonymized to guarantee the privacy of the participants according to the current privacy regulations (EU Regulation GDPR 2016/679).

The study was examined and approved by the Ethical Committee of Liguria Region (361/2022).

## 5 Results

### 5.1 Validation of the Instrument

#### 5.1.1 Content Validation

A total of six experts evaluated the relevance of the items of the questionnaire. All items obtained an I‐CVI ≥ 0.78, except for 5 that obtained I‐CVIs = 0.70. Since the S‐CVI resulted = 0.91, every item was maintained.

#### 5.1.2 Face Validation

A total of 10 possible participants participated in the face validation. In general, none of the questions were considered offensive. We modified the items that were considered unclear to improve the intelligibility by adding the definitions of the words that participants expressed difficulties with.

#### 5.1.3 Psychometric Evaluation

A total of 633 hospital personnel participated in the survey. Most participants were nurses (55%), females (75%), with a mean age of 46 years, who have been working for more than 11 years (54%), currently working in a clinical inpatient unit (65%), with direct interaction with patients (89%). Characteristics of participants are presented in Table 1.

**TABLE 1:** Characteristics of participants (total).

Items	*n* (%)	Mean (SD)	Missing
			*n* (%)
Profession			8 (1.3)
Nurse	345 (55.2)		
Midwife	1 (0.2)		
Physician	117 (18.7)		
Nursing assistant	80 (12.8)		
Other clinical profession (e.g., physiotherapist, dietician, etc.)	32 (5.1)		
Non‐physician graduate health professionals (e.g., biologist, psychologist, etc.)	9 (1.4)		
Pharmacist	1 (0.2)		
Administrative position	18 (2.9)		
Support services	2 (0.3)		
Other	20 (3.2)		
Gender			158 (25.0)
Male	95 (20)		
Female	356 (74.9)		
Rather not answer	24 (5.1)		
Age		46.4 (12)	158 (25.0)
Current hospital of work			167 (26.4)
San Martino Polyclinic Hospital	308 (66.1)		
San Luca Hospital	158 (33.9)		
Years of work in the hospital			158 (25.0)
Less than 1 year	56 (11.8)		
1–5 years	125 (26.3)		
6–10 years	38 (8.0)		
More than 11 years	256 (53.9)		
Current operative unit/working area			31 (4.9)
Clinical inpatient unit	392 (65.1)		
Outpatient services	118 (19.6)		
Hospital services	31 (5.1)		
Administration/management	55 (9.1)		
Support services	6 (1.0)		
Years of work in the current operative unit			158 (25.0)
Less than 1 year	90 (18.9)		
1–5 years	196 (41.3)		
6–10 years	56 (11.8)		
More than 11 years	133 (28.0)		
Hours of weekly work			158 (25.0)
Less than 30 h/week	16 (3.4)		
30–40 h/week	360 (75.8)		
More than 40 h/week	99 (20.8)		
Direct interaction with patients			158 (25.0)
Yes	421 (88.6)		
No	54 (11.4)		
Total participants	633 (100)		

Of these participants, 74.7% (*n* = 473) completed the questionnaire in its entirety. We evaluated the missing values and the floor‐ceiling effect, excluding the demographic and non‐ordinal variables (Table 2). Missing values ranged from 17% (section A, investigating the current operative unit/working area) to 24% (section F, investigating the current hospital). As regards the floor‐ceiling effect, all ranges of possible scoring were used for every item. The items that obtained the highest percentage of score 1 and score 5 were both from section C, investigating communication. In particular, the items respectively asked if “in this operative unit/working area, the staff is afraid to ask questions when something does not seem right” (score 1, 31%) and if “when errors happen in this unit, the staff discuss ways to prevent them from happening again” (score 5, 33.2%).

**TABLE 2:** Missing values, floor‐ceiling effect.

Items	Missing	Floor‐ceiling effect	
		*n* (%)	
	*n* (%)	Score 1	Score 5
SA_Q1	108 (17.1)	24 (4.6)	87 (16.6)
SA_Q2	110 (17.4)	106 (20.3)	26 (5.0)
SA_Q3	110 (17.4)	19 (3.6)	85 (16.3)
SA_Q4	110 (17.4)	35 (6.7)	65 (12.4)
SA_Q5	110 (17.4)	74 (14.1)	17 (3.3)
SA_Q6	110 (17.4)	55 (10.5)	36 (6.9)
SA_Q7	110 (17.4)	60 (11.5)	39 (7.5)
SA_Q8	110 (17.4)	13 (2.5)	121 (23.1)
SA_Q9	110 (17.4)	119 (22.8)	20 (3.8)
SA_Q10	111 (17.5)	23 (4.4)	64 (12.3)
SA_Q11	110 (17.4)	56 (10.7)	54 (10.3)
SA_Q12	110 (17.4)	22 (4.2)	52 (9.9)
SA_Q13	110 (17.4)	31 (5.9)	36 (6.9)
SA_Q14	110 (17.4)	50 (9.6)	34 (6.5)
SA_Q15	110 (17.4)	18 (3.4)	54 (10.3)
SB_Q1	123 (19.4)	24 (4.7)	80 (15.7)
SB_Q2	123 (19.4)	59 (11.6)	11 (2.2)
SB_Q3	123 (19.4)	12 (2.4)	87 (17.1)
SC_Q1	133 (21.0)	3 (0.6)	157 (31.4)
SC_Q2	133 (21.0)	17 (3.4)	166 (33.2)
SC_Q3	133 (21.0)	18 (3.6)	127 (25.4)
SC_Q4	133 (21.0)	13 (2.6)	160 (32.0)
SC_Q5	133 (21.0)	17 (3.4)	95 (19.0)
SC_Q6	133 (21.0)	11 (2.2)	147 (29.4)
SC_Q7	133 (21.0)	156 (31.2)	23 (4.6)
SD_Q1	142 (22.4)	8 (1.6)	73 (14.9)
SD_Q2	142 (22.4)	11 (2.2)	105 (21.4)
SF_Q1	152 (24.0)	28 (5.8)	42 (8.7)
SF_Q2	152 (24.0)	55 (11.4)	19 (4.0)
SF_Q3	152 (24.0)	12 (2.5)	45 (9.4)
SF_Q4	152 (24.0)	35 (7.3)	22 (4.6)
SF_Q5	152 (24.0)	80 (16.6)	11 (2.3)
SF_Q6	152 (24.0)	19 (4.0)	55 (11.4)

We investigated if there were any differences between those who answered the questionnaire in its entirety and those who compiled it only partially (Table 3). There were no significant differences in questionnaire completion based on gender (*p* = 0.72). Both males and females, along with those who chose not to disclose their gender, had almost identical high questionnaire completion rates, indicating that gender did not play a role in completion. The significant difference in completion rates between different occupations (*p* = 0.026) indicated that profession may influence completion. The significant difference between clinical units (*p* = 0.008) suggested that units could impact the likelihood of completing the HSOPS questionnaire.

**TABLE 3:** Differences between complete and incomplete compilations.

Items	Complete	Incomplete	Total	*p*
Profession (*χ* ^2^)				
Nurse	274 (79.4)	71 (20.6)	345 (100)	0.026*
Midwife	1 (100)	0	1 (100)	
Physician	80 (68.4)	37 (31.6)	117 (100)	
Nursing assistant	62 (77.5)	18 (22.5)	80 (100)	
Other clinical profession (e.g., physiotherapist, dietician, etc.)	26 (81.3)	6 (18.8)	32 (100)	
Non‐physician graduate health professionals (e.g., biologist, psychologist, etc.)	6 (66.7)	3 (33.3)	9 (100)	
Pharmacist	0	1 (100)	1 (100)	
Administrative position	9 (50)	9 (50)	18 (100)	
Support services	2 (100)	0	2 (100)	
Other	13 (65)	7 (35)	20 (100)	
Gender (*χ* ^2^)				
Male	95 (100)	0	95 (100)	0.72
Female	354 (99.4)	2 (0.6)	356 (100)	
Rather not answer	24 (100)	0	24 (100)	
Age (*t*)				0.76
Years of work in the hospital (*χ* ^2^)				
Less than 1 year	56 (100)	0	56 (100)	0.84
1–5 years	124 (99.2)	1 (0.8)	125 (100)	
6–10 years	38 (100)	0	38 (100)	
More than 11 years	255 (99.6)	1 (0.4)	256 (100)	
Current operative unit/working area (*χ* ^2^)				
Clinical inpatient unit	324 (82.7)	68 (17.3)	392 (100)	0.008*
Outpatient services	82 (69.5)	36 (30.5)	118 (100)	
Hospital services	22 (71)	9 (29)	31 (100)	
Administration/management	42 (76.4)	13 (23.6)	55 (100)	
Support services	3 (50)	3 (50)	6 (100)	
Years of work in the current operative unit (*χ* ^2^)				
Less than 1 year	90 (100)	0	90 (100)	0.41
1–5 years	194 (99)	2 (1.0)	196 (100)	
6–10 years	56 (100)	0	56 (100)	
More than 11 years	133 (100)	0	133 (100)	
Hours of weekly work (*χ* ^2^)				
Less than 30 h/week	16 (100)	0	16 (100)	0.73
30–40 h/week	358 (99.4)	2 (0.6)	360 (100)	
More than 40 h/week	99 (100)	0	99 (100)	
Direct interaction with patients (*χ* ^2^)				
Yes	419 (99.5)	2 (0.5)	421 (100)	0.61
No	54 (100)	0	54 (100)	

Abbreviations: *t*, *t*‐test for independent sample; *χ* ^2^, chi‐square test. *p value ≤ 0.05.

For the CFA (Table 4), we only considered participants who answered the questionnaire in its entirety. This sample's characteristics overlapped with those of the total sample. The Barlett's test (*p* < 0.001) and the KMO test (*n* = 0.911) both showed that the data and the sample were adequate for factor analysis. Therefore, we performed a CFA based on the construct defined in the original development of the HSOPS 2 (AHRQ 2019a, 2019b; Sorra et al. 2019). The original study defined 10 factors: teamwork; staffing and work pace; organisational learning‐continuous improvement; response to error; supervisor, manager, and clinical leader support for patient safety; communication about error; communication openness; reporting patient safety events; hospital management support for patient safety; handoffs and information exchange. The Shapiro–Wilk test showed that all variables considered in the CFA were not distributed normally (*p* < 0.001). Thus, we used a robust estimator. In particular, since all the items consisted of questions on a five‐point Likert scale, we used the diagonally weighted least square (DWLS) estimator. We then calculated the Cronbach's alpha for every factor to assess the reliability.

**TABLE 4:** Confirmatory factor analysis.

Construct	Number of items	Cronbach's alpha	GFI	CFI	*χ* ^2^/df	SRMR	RMSEA [90% CI]
1. Teamwork	3	0.70	0.974	0.994	1.2	0.06	0.023 [0.012–0.031]
2. Staffing and work pace	4	0.53					
3. Organisational learning—continuous improvement	3	0.70					
4. Response to error	4	0.77					
5. Supervisor, manager, clinical leader support for patient safety	3	0.72					
6. Communication about error	3	0.89					
7. Communication openness	4	0.71					
8. Reporting patient safety events	2	0.82					
9. Hospital management support for patient safety	3	0.69					
10. Handoffs and information exchange	3	0.67					

Abbreviations: CFI, comparative fit index; CI, confidence interval; RMSEA, root mean square error of approximation; SRMR, standardised root mean square residual; χ^2^/df, Chi‐square index/degrees of freedom (normed chi‐square value).

The analysis showed excellent construct fit, with every index taken into consideration that obtained the desirable values. Cronbach's alpha is good or acceptable for most factors (0.7 ≤ *α* < 0.89), except for two factors that were at the threshold of acceptability (0.67 ≤ *α* < 0.69) and one that was slightly lower (*α* = 0.53). The adapted version of HSOPS 2 can therefore be considered a valid and reliable tool to be used in the Italian context.

### 5.2 Cross‐Sectional

The dimensions that achieved higher percentages of positive responses were “teamwork” (68.2%), “supervisor, manager, or clinical leader support” (67.3%), and “communication about error” (64.2%), while those that achieved the lowest percentage of positive responses were “hospital management support for patient safety” (35.2%), “staffing and work pace” (38%), and “response to error” (46.4%) (Table 5).

**TABLE 5:** Composite score.

	For positively worded items, # of “strongly agree” or “agree” responses	For negatively worded items, # of “strongly disagree” or “disagree” responses	Total # of responses to item (excluding missing and “does not apply/don't know responses”)	Percent positive response to item (%)
1. Teamwork				
*Item A1‐positively worded* “In this unit, we work together as an effective team”	341		522	65.3
*Item A8‐positively worded* “During busy times, staff in this unit help each other”	385		519	74.2
*Item A9‐negatively worded* “There is a problem with disrespectful behaviour by those working in this unit”		333	511	65.2
*Average percent positive response across the 3 items* = 68.2%				
2. Staffing and work pace				
*Item A2‐positively worded* “In this unit, we have enough staff to handle the workload”	161		518	31.1
*Item A3‐negatively worded* “Staff in this unit work longer hours than is best for patient care”		105	514	20.4
*Item A5‐negatively worded* “This unit relies too much on temporary, float, or PRN staff”		283	487	58.1
*Item A11‐negatively worded* “The work pace in this unit is so rushed that it negatively affects patient safety”		216	508	42.5
*Average percent positive response across the 4 items* = 38%				
3. Organisational learning—continuous improvement				
*Item A4‐positively worded* “This unit regularly reviews work processes to determine if changes are needed to improve patient safety”	292		512	57
*Item A12‐positively worded* “In this unit, changes to improve patient safety are evaluated to see how well they worked”	296		499	59.3
*Item A14‐negatively worded* “This unit lets the same patient safety problems keep happening”		242	499	48.5
*Average percent positive response across the 3 items* = 54.9%				
4. Response to error				
*Item A6‐negatively worded* “In this unit, staff feel like their mistakes are held against them”		253	501	50.5
*Item A7‐negatively worded* “When an event is reported in this unit, it feels like the person is being written up, not the problem”		234	502	46.6
*Item A10‐positively worded* “When staff make errors, this unit focuses on learning rather than blaming individuals”	275		504	54.6
*Item A13‐negatively worded* “In this unit, there is a lack of support for staff involved in patient safety errors”		165	489	33.7
*Average percent positive response across the 4 items* = 46.4%				
5. Supervisor, manager, or clinical leader support for patient safety				
*Item B1‐positively worded* “My supervisor, manager, or clinical leader seriously considers staff suggestions for improving patient safety”	322		502	64.1
*Item B2‐negatively worded* “My supervisor, manager, or clinical leader wants us to work faster during busy times, even if it means taking shortcuts”		319	497	64.2
*Item B3‐positively worded* “My supervisor, manager, or clinical leader takes action to address patient safety concerns that are brought to their attention”	368		501	73.5
*Average percent positive response across the 3 items* = 67.3%				
6. Communication about error				
*Item C1‐positively worded* “We are informed about errors that happen in this unit”	325		493	65.9
*Item C2‐positively worded* “When errors happen in this unit, we discuss ways to prevent them from happening again”	334		496	67.3
*Item C3‐positively worded* “In this unit, we are informed about changes that are made based on event reports”	286		481	59.5
*Average percent positive response across the 3 items* = 64.2%				
7. Communication openness				
*Item C4‐positively worded* “In this unit, staff speak up if they see something that may negatively affect patient care”	332		488	68
*Item C5‐positively worded* “When staff in this unit see someone with more authority doing something unsafe for patients, they speak up”	260		467	55.7
*Item C6‐positively worded* “When staff in this unit speak up, those with more authority are open to their patient safety concerns”	324		483	67.1
*Item C7‐negatively worded* “In this unit, staff are afraid to ask questions when something does not seem right”		308	489	63
*Average percent positive response across the 4 items* = 63.5%				
8. Reporting patient safety events				
*Item D1‐positively worded* "When a mistake is caught and corrected before reaching the patient, how often is this reported?"	264		446	59.2
*Item D2‐positively worded* "When a mistake reaches the patient and could have harmed the patient, but did not, how often is this reported?"	266		445	59.8
*Average percent positive response across the 2 items* = 59.5%				
9. Hospital management support for patient safety				
*Item F1‐positively worded* “The actions of hospital management show that patient safety is a top priority”	231		465	49.7
*Item F2‐positively worded* “Hospital management provides adequate resources to improve patient safety”	152		464	32.8
*Item F3‐negatively worded* “Hospital management seems interested in patient safety only after an adverse event happens”		106	461	23.0
*Average percent positive response across the 3 items* = 35.2%				
10. Handoffs and information exchange				
*Item F4‐negatively worded* “When transferring patients from one unit to another, important information is often left out”		208	445	46.7
*Item F5‐negatively worded* “During shift changes, important patient care information is often left out”		307	448	68.5
*Item F6‐positively worded* “During shift changes, there is adequate time to exchange all key patient care information”	274		450	60.9
*Average percent positive response across the 3 items* = 58.7%				

As regards gender, the results showed statistically significant differences for the dimensions of “teamwork” (*p* = 0.0013) and “supervisor, manager, or clinical leader support” (*p* = 0.004) with higher percentages of positive responses in males and lower in those who preferred not to declare their gender. As regards the dimensions of “response to error” (*p* = 0.021), “hospital management support for patient safety” (*p* = 0.033), and “handoffs and information exchange” (*p* = 0.0035), higher percentages of positive responses were from females, while the lower percentages were again from those who preferred not to declare their gender.

Statistically significant differences were also found between the various professions. As regards the dimensions of “communication about error” (*p* < 0.001), “communication openness” (*p* = 0.0067), “reporting patient safety events” (*p* = 0.0054), and “handoffs and information exchange” (*p* = 0.017), higher percentages of positive responses were from nursing assistants, while for “hospital management support for patient safety” (*p* = 0.0035), the higher percentages of positive responses were from administrative workers. In all these dimensions, those who gave the lower percentages of positive responses were other healthcare professionals (e.g., midwives, physiotherapists, biologists, etc.).

Finally, we found the majority of statistically significant differences between clinical inpatient units. The dimensions of “organizational learning–continuous improvement” (*p* < 0.001) and “communication about error” (*p* = 0.0005) obtained higher percentages of positive responses from those working in paediatric units and lower ones from those working in critical care. The dimensions of “response to error” (*p* = 0.018) and “supervisor, manager, or clinical leader support” (*p* = 0.013), obtained higher percentages of positive responses from those working in medical units and lower ones again from those working in critical care. Dimension of “reporting patient safety events” (*p* = 0.0036) also obtained the lower percentages of positive responses from those working in critical care while obtaining the higher ones from those working in surgical units. As regards “staffing and work pace” (*p* = 0.014), higher percentages of positive responses were from those working in psychiatric units and lower ones from those working in surgical units, while for the dimension “hospital management support for patient safety” (*p* = 0.039), higher percentages of positive responses were from those working in paediatric units and the lower ones from those working in psychiatric units.

The complete results are reported in Table 6.

**TABLE 6:** Differences between gender, profession, clinical unit, and dimensions 1 to 10.

Sample characteristics		Average percent positive response	Average percent negative/midpoints response	*χ* ^2^	*p*
1. Teamwork					
Gender	Female	69.5	30.5	13.277	0.0013*
	Male	72.9	27.1		
	Rather not answer	50	50		
Profession	Nurse	68.3	31.7	4.95	0.293
	Physician	69.1	30.9		
	Nursing assistant	69.8	30.2		
	Other HP	59.6	40.4		
	Administrative/other	73.7	26.3		
Clinical unit	Medical	67.8	32.2	7.924	0.161
	Surgical	68.7	31.3		
	Combined (medical‐surgical)	63.5	36.5		
	Critical care	66.5	33.5		
	Paediatric	80.4	19.6		
	Psychiatric	67.9	32.1		
2. Staffing and work pace					
Gender	Female	39.5	60.5	4.855	0.088
	Male	34.7	65.3		
	Rather not answer	25.1	74.9		
Profession	Nurse	38.7	61.3	1.276	0.865
	Physician	37.2	62.8		
	Nursing assistant	36.3	63.7		
	Other HP	35.1	64.9		
	Administrative/other	42.2	57.8		
Clinical unit	Medical	34.9	65.1	14.226	0.014*
	Surgical	24.0	76.0		
	Combined (medical‐surgical)	41.2	58.8		
	Critical care	25.1	74.9		
	Paediatric	39.7	60.3		
	Psychiatric	41.6	58.4		
3. Organisational learning‐continuous improvement					
Gender	Female	58.1	41.9	1.763	0.414
	Male	49.2	50.8		
	Rather not answer	51.1	48.9		
Profession	Nurse	57.7	42.3	5.959	0.202
	Physician	48.9	51.1		
	Nursing assistant	58.6	41.4		
	Other HP	44.1	55.9		
	Administrative/other	53.4	46.6		
Clinical unit	Medical	58.1	41.9	31.84	< 0.001*
	Surgical	49.5	50.5		
	Combined (medical‐surgical)	60.3	39.7		
	Critical care	29.6	70.4		
	Paediatric	65.3	34.7		
	Psychiatric	50.1	49.9		
4. Response to error					
Gender	Female	49.1	50.9	7.739	0.021*
	Male	44.5	55.5		
	Rather not answer	30.5	69.5		
Profession	Nurse	47.6	52.4	3.525	0.474
	Physician	41.4	58.6		
	Nursing assistant	52.0	48.0		
	Other HP	40.7	59.3		
	Administrative/other	44.4	55.6		
Clinical unit	Medical	50.7	49.3	13.634	0.018*
	Surgical	41.1	58.9		
	Combined (medical‐surgical)	43.0	57.0		
	Critical care	29.9	70.1		
	Paediatric	31.2	68.8		
	Psychiatric	34.1	65.9		
5. Supervisor, manager or clinical leader support for patient safety					
Gender	Female	68.3	31.7	11.274	0.004*
	Male	70.0	30.0		
	Rather not answer	49.3	50.7		
Profession	Nurse	66.8	33.2	4.544	0.337
	Physician	64.9	35.1		
	Nursing assistant	75.6	24.4		
	Other HP	64.7	35.3		
	Administrative/other	62.9	37.1		
Clinical unit	Medical	74.0	26.0	14.375	0.013*
	Surgical	65.8	34.2		
	Combined (medical‐surgical)	57.8	42.2		
	Critical care	49.5	50.5		
	Paediatric	64.4	35.6		
	Psychiatric	61.6	38.4		
6. Communication about error					
Gender	Female	64.4	35.6	3.125	0.210
	Male	66.2	33.8		
	Rather not answer	54.9	45.1		
Profession	Nurse	68.0	32.0	27.611	< 0.001*
	Physician	58.2	41.8		
	Nursing assistant	73.9	26.1		
	Other HP	41.8	58.2		
	Administrative/other	50.5	49.5		
Clinical unit	Medical	67.0	33.0	22.06	0.0005*
	Surgical	65.9	34.1		
	Combined (medical‐surgical)	59.0	41.0		
	Critical care	41.2	58.8		
	Paediatric	68.9	31.1		
	Psychiatric	56.3	43.7		
7. Communication openness					
Gender	Female	65.2	34.8	0.805	0.669
	Male	59.1	40.9		
	Rather not answer	62.8	37.2		
Profession	Nurse	65.3	34.7	14.185	0.0067*
	Physician	61.0	39.0		
	Nursing assistant	70.7	29.3		
	Other HP	48.7	51.3		
	Administrative/other	51.5	48.5		
Clinical unit	Medical	63.1	36.9	2.675	0.749
	Surgical	61.8	38.2		
	Combined (medical‐surgical)	53.3	46.7		
	Critical care	60.3	39.7		
	Paediatric	56.8	43.2		
	Psychiatric	57.9	42.1		
8. Reporting patient safety events					
Gender	Female	58.8	41.2	5.749	0.056
	Male	66.7	33.3		
	Rather not answer	50.0	50.0		
Profession	Nurse	61.3	38.7	14.67	0.0054*
	Physician	54.7	45.3		
	Nursing assistant	67.5	32.5		
	Other HP	41.9	58.1		
	Administrative/other	55.3	44.7		
Clinical unit	Medical	62.5	37.5	17.54	0.0036*
	Surgical	69.8	30.2		
	Combined (medical‐surgical)	55.8	44.2		
	Critical care	41.7	58.3		
	Paediatric	57.1	42.9		
	Psychiatric	56.9	43.1		
9. Hospital management support for patient safety					
Gender	Female	37.0	63.0	6.8	0.033*
	Male	32.8	67.2		
	Rather not answer	20.7	79.3		
Profession	Nurse	33.2	66.8	15.64	0.0035*
	Physician	30.8	69.2		
	Nursing assistant	46.0	54.0		
	Other HP	29.3	70.7		
	Administrative/other	50.4	49.6		
Clinical unit	Medical	33.9	66.1	11.681	0.039*
	Surgical	36.1	63.9		
	Combined (medical‐surgical)	35.6	64.4		
	Critical care	24.1	75.9		
	Paediatric	41.0	59.0		
	Psychiatric	23.1	76.9		
10. Handsoff and information exchange					
Gender	Female	60.4	39.6	11.296	0.0035*
	Male	57.4	42.6		
	Rather not answer	38.5	61.5		
Profession	Nurse	57.9	42.1	12.033	0.017*
	Physician	53.9	46.1		
	Nursing assistant	70.4	29.6		
	Other HP	49.3	50.7		
	Administrative/other	65.3	34.7		
Clinical unit	Medical	57.6	42.4	9.078	0.106
	Surgical	66.9	33.1		
	Combined (medical‐surgical)	61.1	38.9		
	Critical care	69.1	30.9		
	Paediatric	68.9	31.1		
	Psychiatric	53.4	46.6		

*p value ≤ 0.05.

## 6 Discussion

This study described the current patient safety culture of hospital personnel working in two Italian hospitals. Also, validation results are reported showing a robust reliability and construct validity.

Results highlighted notable differences in positive responses across the different dimensions investigated by the HSOPS instrument. In particular, “teamwork”, “supervisor, manager, or clinical leader support”, and “communication about error” emerged as dimensions with higher percentages of positive responses, highlighting the general positive perceptions associated with communication, collaboration, and leadership within participants' clinical units. On the other hand, the dimensions that received the lower percentages of positive responses were “hospital management support for patient safety”, “staffing and work pace” and “response to error”, highlighting an overall negative perception of several of the essential organisational components of a strong patient safety culture. These findings are similar to those obtained by multiple studies worldwide (Albaalharith and A'aqoulah 2023; Camacho‐Rodríguez et al. 2022; Azyabi, Karwowski, and Davahli 2021) and also a previous Italian study (Bagnasco et al. 2011). Although some studies may have used different instruments to assess patient safety culture, in fact, the dimensions investigated can be considered largely overlapping. These results are particularly interesting since dimensions such as good teamwork, leadership, and communication within the clinical unit are recognised as notably impactful on patient safety, as they can encourage collaboration aimed at achieving common safety goals within a clinical unit (Azyabi, Karwowski, and Davahli 2021). At the same time, more negative perceptions towards organisational factors, such as staffing or patient safety in general, are suggested to be associated with issues such as missed nursing care, adverse patient safety events, and job‐related stress (Alanazi et al. 2023; Vikan et al. 2023; Zabin et al. 2023; Hessels et al. 2019).

Besides the overall perceptions, it is to notice that many studies focusing on patient safety culture worldwide highlighted differences between positive or negative responses in the different dimensions and various characteristics of participants, such as gender, profession, or working units (Albaalharith and A'aqoulah 2023; Camacho‐Rodríguez et al. 2022; Azyabi, Karwowski, and Davahli 2021; Willmott and Mould 2018; Bagnasco et al. 2011).

In our study, we also identified statistically significant differences in many dimensions. As regards the dimensions that obtained higher percentages of positive response, we identified differences between gender, profession, and clinical inpatient units. In particular, positive responses regarding the “Teamwork” dimension were especially retrieved from males, while those regarding “Communication about error” were especially from nursing assistants and healthcare professionals working in paediatric units. Those regarding “Supervisor, manager, or clinical leader support” were especially from males and healthcare professionals working in medical units.

On the other hand, as regards the dimensions that obtained a lower percentage of positive responses, results showed that females and healthcare professionals working in medical units obtained higher percentages of positive responses as regards “response to error”, while administrative workers and healthcare professionals working in paediatric units tend to have more positive perceptions in terms of “hospital management support for patient safety”, indicating a greater perception of supportive management practices in these professional figures or settings. On the contrary, more negative perceptions seem to prevail in those who preferred not to declare their gender and those working in critical care as regards “response to error”, and in other healthcare professionals (such as midwives, physiotherapists, etc.) and in those working in the psychiatric units as regards “hospital management support for patient safety”. In the dimension of “staffing and work pace” higher percentages of positive responses were observed amongst professionals working in psychiatric units, indicating a perceived adequacy of staffing levels and manageable work pace in these settings. Conversely, professionals in surgical units reported lower percentages of positive response in this dimension, suggesting potential challenges related to staffing shortages or excessive workloads, which can compromise patient safety, including inpatient mortality. This reflects variations in workload, staffing levels, and practice dynamics across different clinical specialties, emphasising the need for tailored interventions to address specific challenges faced by each unit (Catania et al. 2024).

We found other differences between the sample's characteristics and other dimensions that are interesting to note. For instance, we found that the dimension “Handoffs and Information Exchange” obtained higher percentages of positive responses from females and from nursing assistants, while lower ones from those who preferred not to declare their gender and other healthcare professionals (e.g., midwives, physiotherapists, biologists, etc.). Higher percentages of positive responses from nursing assistants and lower ones from other healthcare professionals are also shown as regards the dimensions of “communication openness” and “reporting patient safety events”. Lastly, we found that healthcare professionals working in critical area obtained lower percentages of positive responses in other dimensions, such as “organizational learning—continuous improvement” and “reporting patient safety events”.

These findings offer an overview of the dynamics within the healthcare environment that influence patient safety culture. For instance, the fact that positive responses for specific dimensions are more prevalent amongst certain professions or certain clinical inpatient units suggests potential disparities in training or experiences influencing perceptions of patient safety and indicates the need for targeted interventions to enhance the working environment (Mistri, Badge, and Shahu 2023; Azyabi, Karwowski, and Davahli 2021). Finally, identifying differences in responses regarding reporting adverse events is crucial for fostering a culture of patient safety, as such data can help identify specific barriers to reporting and develop strategies to encourage greater transparency and accountability in addressing errors and improving overall healthcare quality. Indeed, the differences between professions or clinical settings need to be addressed to enhance a shared culture of safety as recommended. The NHS defines a positive safety culture as an environment that is collaboratively crafted, created, and nurtured so that everybody (individual staff, teams, patients, service users, families, and carers) can flourish to ensure safe care (NHS 2022). This can be achieved through continuous learning and improvement of safety risks, supportive and psychologically safe teamwork, and enabling and empowering speaking up by all (NHS 2022).

In conclusion, these findings provide a comprehensive overview of the challenges and opportunities within the healthcare sector, offering a robust foundation for the implementation of targeted interventions aimed at improving clinical practice, the working environment, and patient safety.

### 6.1 Strength and Limitations of the Work

This study has some limitations. In fact, the study involved only two Italian hospitals in the north of Italy, and this could imply that other Italian regions, if investigated, might have produced different results. Moreover, although we retrieved almost 75% of the questionnaires filled entirely, which is a satisfying result, it is to note that we also had a percentage of missing data referred to incomplete answers (i.e., participants that gave their consent without completing the questionnaire). Moreover, we included all hospital personnel that accepted to participate in the study. This might include newly recruited hospital personnel, who may still not have a thorough understanding of hospital policies, which could potentially impact the generalizability of the results. Despite this, we were able to validate the updated version of one of the most utilised tools to assess patient safety culture in hospitals, which allowed us to compare our results with studies from all over the world and to place our results within the international landscape. This is an important goal; however, it is to notice that for one of the dimensions (“staffing and work pace”), our Cronbach's alpha value was quite low. This was possibly due to heterogeneous sample characteristics. Moreover, we were able to involve many different professionals and investigate many different clinical units and settings, which allowed us to give a more comprehensive view on patient safety culture.

### 6.2 Recommendations for Further Research

Future research should focus on gaining a deeper understanding of the dynamics highlighted by this study, their implications, and the underlying factors driving these disparities. For example, patient safety logistics should be investigated to understand at the hospital and unit level the activities carried out for patient safety, including incident reporting and learning, application and monitoring of safe practices, and continuous professional education on risk prevention. Such exploration could shed light on the underlying reasons for the observed differences and offer insights into how to best foster a positive and inclusive organisational culture. By addressing these nuances, healthcare organisations can work towards creating environments that promote effective communication, collaboration, and leadership for all individuals, regardless of their demographic or professional background, by informing tailored strategies for promoting a culture of safety.

### 6.3 Implications for Policy and Practice

Effective management support seems vital for fostering a culture of safety within healthcare organisations, and our findings underscore the importance of proactive management practices in promoting patient safety and enhancing healthcare professionals' confidence in the delivery of safe care. Managers and administrators should prioritise resource allocation, staffing levels, and supportive policies to systemically address challenges and cultivate a culture of safety across clinical settings. Moreover, the results of this study suggest that investigating hospital personnel's perception towards patient safety culture may help to develop tailored interventions to face specific challenges experienced by professionals in different clinical units. For instance, initiatives aimed at improving staffing levels and management support may be particularly beneficial for those working in units that experience workload‐related concerns. Similarly, strategies to enhance communication and collaboration between frontline staff and management may be warranted in units where perceptions of management support are lower. Finally, results suggest that response to error should be addressed as a key step to manage the consequences of adverse outcomes for patients, families, and for healthcare workers. Patients' involvement for safety and protection for healthcare workers who openly disclose an incident have been recently addressed by the WHO during the World Patient Safety Day in 2023 (O'Hara and Canfield 2024) and in the patient safety rights charter (WHO 2024).

## 7 Conclusions

This study sheds light on the multifaceted nature of hospital personnel's perceptions of patient safety, elucidating variations influenced by gender, profession, and clinical context. Understanding these nuances is crucial for developing, integrating, and testing targeted interventions to enhance patient safety culture across diverse healthcare settings. Also, patient safety culture should be addressed within the undergraduate programs and at higher educational level programs, including continuous professional development initiatives. Involving patients and their caregivers could additionally improve the effectiveness of patient safety initiatives.

## Author Contributions

All authors made substantial contributions to the conception or design of the work or the acquisition, analysis, or interpretation of data for the work and drafted the work or reviewed it critically for important intellectual content and gave final approval of the version to be published and agreed to be accountable for all aspects of the work in ensuring that questions related to the accuracy or integrity of any part of the work are appropriately investigated and resolved. **Annamaria Bagnasco:** conceptualization, writing – review and editing, supervision, and project administration. **Gianluca Catania:** conceptualization, methodology, validation, formal analysis, investigation, data curation, writing – original draft, and visualisation. **Michele Tancredi Loiudice:** conceptualization, methodology, investigation, data curation, writing – review and editing, and visualisation. **Tommaso Bellandi:** conceptualization, methodology, investigation, data curation, writing – review and editing, and visualisation. **Bruno Cavaliere:** conceptualization, writing – review and editing, and supervision. **Sara Carzaniga:** conceptualization, methodology, investigation, data curation, writing – review and editing, and visualisation. **Flavia Cardinali:** conceptualization, methodology, investigation, data curation, writing – review and editing, and visualisation. **Milko Zanini:** conceptualization, writing – review and editing, and supervision. **Loredana Sasso:** conceptualization, writing – review and editing, and supervision. **the Working Group Study:** methodology, data curation, writing – original draft, and visualisation.

## Ethics Statement

The study was approved by the Ethical Committee of Liguria Region (#361/2022).

## Conflicts of Interest

The authors declare no conflicts of interest.

## Peer Review

The peer review history for this article is available at https://www.webofscience.com/api/gateway/wos/peer‐review/10.1111/jan.16770.

## Supporting information


Data S1.

## Data Availability

Data available on request from the authors.

## Appendix 1

Nicola Pagnucci^5^

Michela Calzolari^1^

Francesca Napolitano^1^

Davide Ulivieri^6^

Spartaco Mencaroni^7^

Luca Lavazza^7^

^5^Dipartimento di Ricerca Traslazionale e delle Nuove Tecnologie in Medicina e Chirurgia, Università degli Studi di Pisa, Pisa, Italy

^6^Referente Gestione della qualità dell'assistenza e analisi dei bisogni formativi, Ospedale IRCCS Policlinico San Martino, Genova, Italy

^7^Azienda USL Toscana Nord Ovest, Cittadella della Salute, Lucca, Italy
